# Possible associations between plasma fibroblast growth factor 21 levels and cognition in bipolar disorder

**DOI:** 10.1002/npr2.12102

**Published:** 2020-04-08

**Authors:** Favour Omileke, Sayuri Ishiwata, Junko Matsuo, Fuyuko Yoshida, Shinsuke Hidese, Kotaro Hattori, Hiroshi Kunugi

**Affiliations:** ^1^ Department of Mental Disorder Research National Institute of Neuroscience National Centre of Neurology and Psychiatry (NCNP) Tokyo Japan; ^2^ Medical Genome Center NCNP Tokyo Japan

**Keywords:** bipolar disorder, cognitive impairment, FGF21

## Abstract

Bipolar disorder (BD) is a mental disorder characterized by extreme changes in mood polarity. It is also characterized by cognitive and metabolic dysfunctions. Fibroblast growth factor 21 (FGF21) is an endocrine protein that has a multifaceted function such as glucose and lipid regulation in the periphery, and neuroprotection and induction of synaptic plasticity in the central nervous system. Previous studies reported inconsistent results concerning peripheral FGF21 levels in patients with BD. In this study, we compared plasma FGF21 levels between 26 patients with BD and 51 healthy controls using a human FGF21 ELISA Kit. There was no significant difference in plasma FGF21 levels between the patients and controls. We found significant positive correlations between plasma FGF21 levels and some cognitive parameters (word association and motor speed). If our results are replicated that higher peripheral FGF21 may be associated with better cognitive performance in patients with BD.

## Abstract 2

We compared plasma FGF21 levels between 26 bipolar disorder patients and 51 healthy controls. Although FGF21 concentrations were not different between the patients and controls, we found significant positive correlations between the levels and some cognitive parameters.

## Graphical abstract

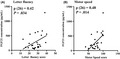

## 1 INTRODUCTION

Bipolar Disorder (BD) is a mental disorder characterized by extreme fluctuations in mood polarity ranging from manic to melancholic depressed states.^1^ It is also characterized by several types of dysfunctions such as cognitive, neurological, and metabolic dysfunctions.^2^ As a result, BD has a complex pathological course, which often results in less favorable treatment response and adverse outcomes.^3^ Therefore, to treat BD effectively and attain overall better outcomes, it would be necessary to generate a form of treatment, in addition to mood stabilizers that addresses cognitive, neurological, and metabolic dysfunctions.

Fibroblast growth factor 21 (FGF21) belongs to a family of structurally related proteins that play an active role in homeostasis, cell proliferation, and survival.^4^ FGF21 and other members of the FGF family of ligands have a low level of binding to heparin/heparan surface and therefore circulate as hormones.^5^ FGF21 mediates its effects through cell surface receptors such as the FGF receptors, which are tyrosine kinases that form a noncovalent complex with the transmembrane coreceptor *β*‐Klotho.^6^ FGF21 also has central and peripheral stress‐responsive roles where it regulates lipid and glucose homeostasis as a response to nutrient stress by increasing energy expenditure and improving insulin sensitivity and lipid parameters.^7^, ^8^ In addition, FGF21 mediates adaptive responses to tissue injury and repair in pathologically stressful conditions.^9^ It exerts therapeutic effects through reducing inflammation and oxidative stress and promoting tissue repair. Moreover, in the central nervous system (CNS), FGF21 has a neuroprotective function where it acts through different signaling cascades to induce synaptic plasticity, increase synaptic density and dendritic spines.^10^ It also aids in regulating the circadian rhythm and induces sympathetic nervous activity to modulate energy expenditure.^11^ In the periphery, FGF21 is secreted mainly in the liver; however, it is also secreted in both brown and white adipocytes, the pancreas, and skeletal muscles.^12^ FGF21 plays some roles in brain. For example, intracerebroventricular infusion of FGF21 increased hepatic insulin sensitivity and metabolic rate in rats with diet‐induced obesity.^13^ Furthermore, recombinant FGF21 treatment corrects high‐fat diet–induced cognitive impairment and anxiety‐like behavior in mice.^14^

Due to FGF21’s multifaceted roles, this protein may play a role in the pathophysiology of psychiatric disorders such as BD. Liu et al,^15^ reported a negative association between cerebrospinal fluid (CSF) FGF21 levels and scores on Beck's depression index (BDI) in male nonclinical volunteers. In a longitudinal study, Chang et al,^1^ revealed that there was no significant difference in plasma FGF21 levels between depressed BD patients and healthy controls at baseline. They also reported a significant increase in patients’ FGF21 levels after treatment with valproate (VPA) and fluoxetine for 12 weeks. However, this increase was associated with less symptom reduction during the treatment. On the other hand, Hu et al,^16^ demonstrated that serum FGF21 levels in manic BD patients were significantly higher at baseline than those of controls. After a 4‐week treatment with 2nd generation antipsychotic drugs, there was a significant reduction in FGF21 serum levels, although the concentration was still higher than those of controls.

Seeing as these inconsistent results require further investigation, the present study aimed to examine plasma FGF21 levels in patients with BD relative to healthy controls. In addition, we focused on the potential relationship between FGF21 and cognitive functions in BD. To our knowledge, there is no other study that has examined the possible relationships between FGF21 levels and cognitive functions in BD.

## 2 METHODS

### 2.1 Participants

Participants were 26 patients with BD (14 females, mean age = 44.5 years, SD = 14.52) and 51 healthy controls (26 females, mean age = 45.7 years, SD = 12.8). Participants were recruited through local advertisements and the National Centre of Neurology and Psychiatry's (NCNP) website and hospital (Tokyo, Japan). Trained psychiatrist and psychologists screened all participants using the Mini International Psychiatric Interview (MINI),^17^ Japanese edition.^18^ A consensus was made by at least two psychiatrists according to the Diagnostic and Statistical Manual of Mental Disorders, 4th edition (DSM‐IV) criteria^19^ and medical records, if available. We excluded individuals with prior medical history of CNS diseases, severe head injury, or substance abuse/dependence. We also excluded individuals who had or do currently have regular contact with psychiatrists or a history of psychotropic drug use from the control group. We conducted the present study in accordance with the declaration of Helsinki, and the protocol was approved by the ethics committee at NCNP (no. 305). All participants provided written informed consent.

### 2.2 Materials

The plasma samples used in this experiment are nonfasting samples. The patient samples are from those who had undergone pharmacotherapy with drugs such as antipsychotics, mood stabilizers, and antidepressant drugs. We determined FGF21 levels using human FGF21 ELISA Kit (abcam) according to the manufacturer's instructions. The limit of detection was 3.3 pg/ mL. The antibodies used in this ELISA kit were specific for human FGF21. We measured the absorbance level at 450 nm. We examined plasma FGF21 levels over the course of two days to ensure that our methods are replicable. We calculated the mean between two 96‐well plates to verify the replicability of our results. We chose 30% as the threshold for the coefficient of variation (CV), and we omitted participants that had a CV value above our threshold value. Thus, one participant was omitted from our analysis.

### 2.3 Clinical assessment

To assess cognitive function in our participants, we used the Brief Assessment of Cognition in Schizophrenia (BACS).^20^ Depressive symptoms were assessed with the Hamilton Rating Scale for Depression (HAM‐D).^21^

### 2.4 Statistical analysis

We used the Shapiro‐Wilk test to examine whether our data (plasma FGF21 levels) were normally distributed. The test revealed skewed histograms. Therefore, we chose the medians and interquartile ranges and employed an independent nonparametric test, Mann‐Whitney *U* to compare the plasma samples between diagnostic groups. We assessed the relationships between FGF21’s plasma concentration and our selected variables (age, BMI, and HAM‐D, BACS) using Spearman's correlation. Chi‐square test was used for comparison of sex. We employed SPSS 25.0 (SPSS Inc) statistical package to analyse our data. A two‐tailed *P*‐value result of <.05 was deemed significant.

## 3 RESULTS

**Figure 1:**
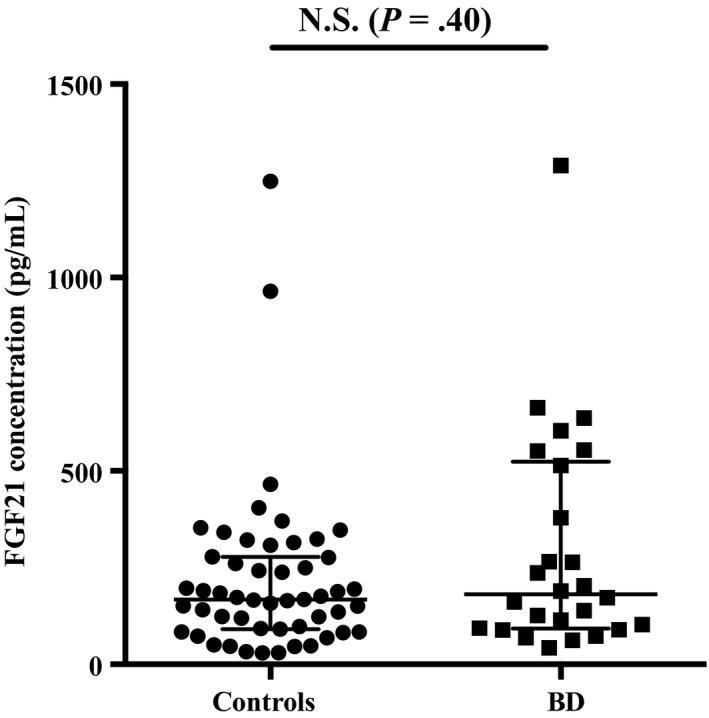
FGF21 concentration in patients and controls. This figure displays a scatter plot of the FGF21 concentration in bipolar patients and controls

**Figure 2:**
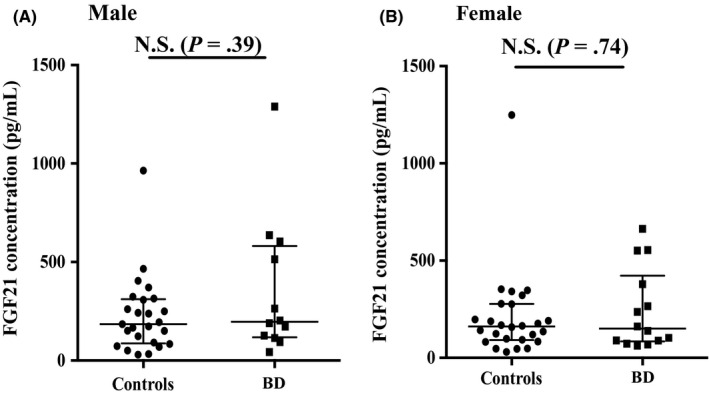
FGF21 concentration in male or female patients and controls. This figure displays a scatter plot of the FGF21 concentration in bipolar patients and controls in males (A) and females (B)

**Figure 3:**
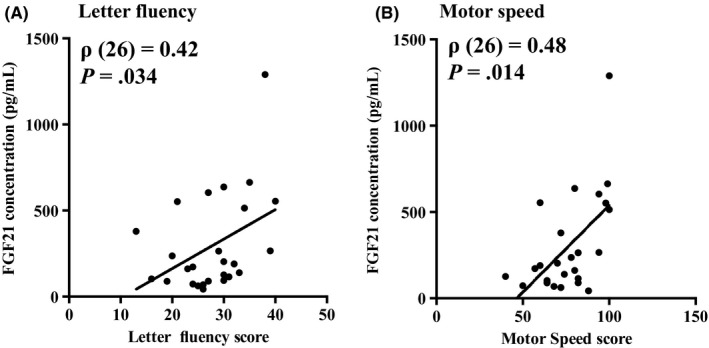
Association between plasma FGF21 concentration and cognitive function in patients. This figure displays a significant positive correlation between plasma FGF21 concentration and letter fluency (A) or motor speed (B) in bipolar patients

Table 1 presents participants’ demographic and clinical data. There was no significant difference in plasma FGF21 levels between patients and controls (*U* = 585, *P* = .40) (Figure 1). Plasma FGF21 levels were not significantly correlated with age or BMI either in patients or in controls (all *P* > .05) (data not shown). There was no significant difference in FGF21 levels between male and female for the two diagnostic groups (Male: *U* = 123, *P* = .39, Female: *U* = 170, *P* = .74) (Figure 2A,B). There was no significant correlation between plasma FGF21 levels and BACS composite score for both patients, ρ(26) = −0.13, *P* > .05 and controls, ρ(38) = −0.05, *P* > .05 (data not shown). However, we observed that plasma FGF21 levels were positively correlated with two subscales of the BACS, namely letter fluency, ρ(26) = 0.42, *P* = .034 (Figure 3A) and motor speed, ρ(26) = 0.48, *P* = .014 (Figure 3B).

**Table 1:** Demographic and clinical data with BACS scores

	BD	Controls	Statistics (*P*‐value)
N	26	51	
	Type I: 9, Type II: 17		
Male/female (N)	12/14	25/26	χ^2^ = 0.24, *df* = 1, *P* = .62 (Pearson's chi‐square test)
Age (Median)	44.5 (33.5‐57.3)	48.0 (34.0‐57.0)	.81 (Mann‐Whitney *U* test)
Duration of illness	10.0 (4.8‐15.5)		
Age at onset	27.5 (20.8‐35.8)		
Number of hospitalization	0 (0‐1.3) (N = 22)		
Education	14.0 (12.0‐16.0)		
BACS			
Verbal memory	47.0 (41.8‐57.0) (N = 26)	47.0 (35.8‐55.0) (N = 38)	.16 (Mann‐Whitney *U* test)
Working memory	21.5 (17.8‐25.0) (N = 26)	20.50 (17.0‐24.0) (N = 38)	.70 (Mann‐Whitney *U* test)
Motor speed	76.0 (64.0‐89.5) (N = 26)	77.50 (68.0‐96.0) (N = 38)	.43 (Mann‐Whitney *U* test)
Category fluency	20.5 (18.8‐23.0) (N = 26)	21.0 (17.8‐25.0) (N = 38)	.94 (Mann‐Whitney *U* test)
Letter fluency	28.0 (23.8‐32.3) (N = 26)	26.0 (22.5‐31.0) (N = 38)	.41 (Mann‐Whitney *U* test)
Attention	62.0 (54.8‐72.3) (N = 26)	64.00(58.5‐67.3) (N = 38)	.87 (Mann‐Whitney *U* test)
Executive function	18.5 (16.8‐19.0) (N = 26)	18.00 (15.0‐19.0) (N = 38)	.51 (Mann‐Whitney *U* test)
Composite score	−0.05 (−0.6 to 0.6) (N = 26)	0.09 (−0.5 to 0.6) (N = 38)	.86 (Mann‐Whitney *U* test)

Note

This table presents our participants’ demographic information and their scores on the BACS questionnaire. Statistical analysis was performed using Pearson's chi‐square test or Mann‐Whitney *U* test.

Since the patients were medicated, we showed the drug information in Table 2. We examined the correlation between FGF21 concentrations, and chlorpromazine (CP) equivalent value, typical CP equivalent value, atypical CP equivalent value or imipramine equivalent value. However, we found no significant association between any of the equivalent value and FGF concentrations (Data not shown).

**Table 2:** Drug information in bipolar patinets

No.	Typical antipsychotics	Atypical antipsychotics	Antidepressant	Mood stabilizer	Lithium
1	1	0	0	0	0
2	0	1	0	1	0
3	1	1	0	0	0
4	0	0	0	1	1
5	0	0	1	0	0
6	0	0	1	0	1
7	0	0	1	0	0
8	0	1	1	0	1
9	0	0	1	0	0
10	0	0	1	No information	0
11	0	0	1	0	1
12	0	0	1	0	0
13	0	0	0	0	0
14	1	1	1	0	0
15	0	0	0	0	0
16	0	1	1	1	0
17	0	0	0	0	0
18	0	0	0	0	0
19	0	0	0	0	0
20	0	0	0	0	0
21	0	0	0	0	0
22	0	0	1	1	0
23	0	0	0	0	1
24	1	0	1	1	0
25	0	0	1	1	0
26	0	0	1	0	0

0, None; 1, Took medicines.

## 4 DISCUSSION

The present study compared plasma FGF21 levels between patients with BD and controls and examined the potential relationship between cognitive parameters and FGF21 in BD. There was no significant difference in plasma FGF21 levels between patients and controls. However, we found significant positive correlations between plasma FGF21 levels and two subscales of the Brief Assessment of Cognition in Schizophrenia (BACS) in patients with BD; letter fluency and motor speed, thereby indicating that higher plasma FGF21 levels are potentially associated with improved cognitive functions in patients with BD. Plasma FGF21 levels were not correlated with depression severity assessed with HAM‐D.

We found no significant difference in plasma FGF21 levels between patients and controls, which is similar to Chang et al,^1^ who also reported that there was no significant difference in plasma FGF21 levels between depressed BD patients at baseline (ie, prior to treatment) and healthy controls. On the other hand, Hu et al,^16^ reported that serum FGF21 levels in manic BD patients were significantly higher than those of controls. The inconsistency between this result and ours may be due to the fact that the majority of our patients were depressed. It is probable that FGF21 levels may differ depending on BD’s polarity. Moreover, different peripheral samples (ie, plasma vs serum) may have contributed to the inconsistent results.

We found that plasma FGF21 levels correlated with cognitive functions in BD. Letter fluency and motor speed are indicative of memory function, attention and executive function, which are the domains of cognition^22^ that are mainly impaired in BD. Patients with BD exhibit impaired declarative memory, executive function, and attention as well as psychomotor retardation relative to healthy controls. These impairments are present in both types of BD, but may differ in intensity depending on the type of BD.^23^

Our findings may suggest that FGF21 may play a potential role in attenuating cognitive impairment. This is feasible, considering FGF21’s neuroprotective role. Leng et al,^24^ sought to identify the molecular mechanisms involved in the synergistic neuroprotective effects induced by mood stabilizers (lithium and VPA) in the rat brain. They found that FGF21 mRNA was robustly induced in hippocampal and cortical neurons by cotreatment with mood stabilizers and that** **exogenous FGF21 completely blocked glutamate‐induced excitotoxicity and apoptosis. Furthermore, FGF21’s neuroprotective benefits were associated with a rapid increase in phosphorylation in Akt1 and other signaling pathways, which may aid in amelioratory cognitive decline. In addition, Sa‐nguanmoo et al,^10^ reported that plasma FGF21 treatment restored brain mitochondrial function by reducing reactive oxygen species (ROS) production and brain oxidative stress levels in obese insulin‐resistant male rats. This resulted in decreased brain cell apoptosis, restored hippocampal synaptic plasticity, and restored dendritic spine, leading to slowing down cognitive decline. Furthermore, Wang et al,^14^ also demonstrated that recombinant FGF21 (rFGF21) corrected cognitive impairment and anxiety‐like behavior as well as glucose metabolic disorder and hyperlipidaemia in high‐fat diet (HFD) obese mice. Moreover, rFGF21 was found to reduce systemic pro‐inflammation through mechanisms involving Akt1 signaling in these obese mice. Together, these animal experiments suggest that FGF21 does not only have a therapeutic role in regulating metabolic dysfunction in BD, but it may also have a neuroprotective role, which may play a role in countering cognitive dysfunction.

There are several limitations in the present study; firstly, we had a small sample size which may have increased the risk of obtaining a type II error. In addition, our patient sample suffered from milder forms of BD, which may have also increased the risk of obtaining a type II error. Secondly, the majority of patients were medicated, which may have influenced plasma FGF21 levels. Thirdly, cognition is a broad subject that comprises of multiple dimensions. The main dimensions that are impaired in BD are attention, memory and executive function.^25^ The fact that we focused on general cognition is a shortcoming of this study, as FGF21’s role in ameliorating cognition may not be applicable to all aspects of cognition.

Future studies with a larger sample and nonmedicated patients are necessary to address these limitations. In this study, the majority of the patients were medicated with different kinds of antipsychotic and mood stabilizer drugs, which may have influenced cognitive functions. To avoid the effect of differential medication status, studies controlling for medication will be required. Also, it is necessary to examine the relationship between FGF21 and cognition in more detail. Indeed, BACS does not cover all domains of cognition. Future studies should use a more comprehensive test battery such as Measurement and treatment research improve cognition in schizophrenia (MATRICS).

In addition, studies are required to explore the mechanisms underlying FGF21’s actions; therefore, future research could examine the mechanisms through which FGF21 acts to improve cognition in BD.

In conclusion, the present study examined the possible relationship between plasma FGF21 and BD. There was no significant difference in plasma FGF21 levels between patients with BD and healthy controls. However, we found that higher plasma FGF21 levels were associated with better cognitive performance in some cognitive parameters (letter fluency and motor speed). Our study is limited by the small sample size; therefore, future replication studies using a larger sample size are necessary to confirm the relationship between FGF21 and cognition. These studies may aid in generating new strategies in the treatment of BD.

## CONFLICTS OF INTEREST

None.

## AUTHOR CONTRIBUTION

FO, SI, and HK designed the study. FO performed the biochemical assays and analyzed the data. HK, SH, and KH recruited and assessed the patients and collected the plasma samples. JM, SH, and HK evaluated the clinical assessments. FY managed the plasma samples. FO, SI, and HK wrote the paper.

## DATA REPOSITORY

Additional supporting information may be found online in the Supporting Information section.

## APPROVAL OF THE RESEARCH PROTOCOL BY AN INSTITUTIONAL REVIEWER BOARD

This study was approved by National Center of Neurology and Psychiatry ethics committee.

## INFORMED CONSENT

Written informed consent was obtained from all subjects.

## REGISTRY AND THE REGISTRATION NO. OF THE STUDY/TRIAL

N/A.

## ANIMAL STUDIES

N/A.

## Supporting information

Supplementary Material
